# Immune Protection against *Trypanosoma cruzi* Induced by TcVac4 in a Canine Model

**DOI:** 10.1371/journal.pntd.0003625

**Published:** 2015-04-08

**Authors:** José E. Aparicio-Burgos, José A. Zepeda-Escobar, Roberto Montes de Oca-Jimenez, José G. Estrada-Franco, Alberto Barbabosa-Pliego, Laucel Ochoa-García, Ricardo Alejandre-Aguilar, Nancy Rivas, Giovanna Peñuelas-Rivas, Margarita Val-Arreola, Shivali Gupta, Felix Salazar-García, Nisha J. Garg, Juan C. Vázquez-Chagoyán

**Affiliations:** 1 Escuela Superior de Apan-Universidad Autónoma del Estado de Hidalgo, Apan Hidalgo, México; 2 Centro de Investigación y Estudios Avanzados en Salud Animal, Universidad Autónoma de Estado de México, Toluca, México; 3 Laboratorio Estatal de Salud Pública del Instituto Salud del Estado de México, Toluca, México; 4 Laboratorio de Entomología, Departamento de Parasitología, Escuela Nacional de Ciencias Biológicas del Instituto Politécnico Nacional, México City, México; 5 Hospital General de Zona No. 2, Instituto Mexicano del Seguro Social, Irapuato, México; 6 Department of Microbiology and Immunology, University of Texas Medical Branch (UTMB), Galveston, Texas, United States of America; 7 Department of Pathology, and Faculty of the Institute for Human Infection and Immunity, and the Sealy Center for Vaccine Development, University of Texas Medical Branch (UTMB), Galveston, Texas, United States of America; Universidade Federal de Minas Gerais, BRAZIL

## Abstract

Chagas disease, caused by *Trypanosoma cruzi*, is endemic in southern parts of the American continent. Herein, we have tested the protective efficacy of a DNA-prime/*T*. *rangeli*-boost (TcVac4) vaccine in a dog (*Canis familiaris*) model. Dogs were immunized with two-doses of DNA vaccine (pcDNA3.1 encoding TcG1, TcG2, and TcG4 antigens plus IL-12- and GM-CSF-encoding plasmids) followed by two doses of glutaraldehyde-inactivated *T*. *rangeli* epimastigotes (TrIE); and challenged with highly pathogenic *T*. *cruzi* (*Sylvio*X10/4) isolate. Dogs given TrIE or empty pcDNA3.1 were used as controls. We monitored post-vaccination and post-challenge infection antibody response by an ELISA, parasitemia by blood analysis and xenodiagnosis, and heart function by electrocardiography. Post-mortem anatomic and pathologic evaluation of the heart was conducted. TcVac4 induced a strong IgG response (IgG2>IgG1) that was significantly expanded post-infection, and moved to a nearly balanced IgG2/IgG1 response in chronic phase. In comparison, dogs given TrIE or empty plasmid DNA only developed high IgG titers with IgG2 predominance in response to *T*. *cruzi* infection. Blood parasitemia, tissue parasite foci, parasite transmission to triatomines, electrocardiographic abnormalities were significantly lower in TcVac4-vaccinated dogs than was observed in dogs given TrIE or empty plasmid DNA only. Macroscopic and microscopic alterations, the hallmarks of chronic Chagas disease, were significantly decreased in the myocardium of TcVac4-vaccinated dogs. We conclude that TcVac4 induced immunity was beneficial in providing resistance to *T*. *cruzi* infection, evidenced by control of chronic pathology of the heart and preservation of cardiac function in dogs. Additionally, TcVac4 vaccination decreased the transmission of parasites from vaccinated/infected animals to triatomines.

## Author Summary

Chagas disease, is an illness caused by *Trypanosoma cruzi*, is endemic in southern parts of the American continent. We tested the protective efficacy of a DNA-prime/*T*. *rangeli*-boost (TcVac4) vaccine in a dog (*Canis familiaris*) model. Dogs were immunized with two-doses of DNA vaccine followed by two doses of inactivated *T*. *rangeli* epimastigotes (TrIE) and challenged with a highly pathogenic strain of *T*. *cruzi*. TcVac4 induced a protective antibody response in comparison with dogs from the control groups. Blood parasite burden, parasite transmission to triatomines and electrocardiographic abnormalities were significantly lower in TcVac4-vaccinated dogs than was observed in dogs given TrIE or empty plasmid DNA only. Macroscopic and microscopic alterations were significantly decreased in the myocardium of TcVac4-vaccinated dogs. We conclude that TcVac4 induced immunity was beneficial in providing resistance to *T*. *cruzi* infection, evidenced by control of chronic heart damage and preservation of cardiac function in dogs. Additionally, TcVac4 vaccination decreased the transmission of parasites from vaccinated/infected animals to triatomines.

## Introduction

*Trypanosoma cruzi* (*T*. *cruzi*) is a pathogenic protozoan that belongs to the *Trypanosomatidae* family. It is the etiologic agent of Chagas disease [1]. Approximately, 5% of the infected humans develop a lethal acute infection, and 30–40% progress to a chronic debilitating illness of the cardiac system, characterized by clinically irreversible and progressive myocardial hypertrophy and tissue destruction that eventually leads to heart failure [1]. It is an important health issue in most of the Latin American countries and due to human migration; it has become an important health issue in the United States and Europe [2]. Vector control programs have not been able to completely prevent parasite transmission [3]; the available anti-parasite drugs are not sufficiently safe or effective [4, 5]; and no vaccines are currently available.

Several investigators have shown the potential utility of *T*. *cruzi* surface antigens as vaccine candidates in mice and dogs (reviewed in [6, 7]). Our group has performed computational screening of *T*. *cruzi* sequence databases reported in GenBank, and identified genes encoding glycosylphosphatidylinositol (GPI)-anchored proteins TcG1, TcG2 and TcG4 as potential vaccine candidates. These antigens were chosen after an unbiased computational/bioinformatics screening of the *T*. *cruzi* genome sequence database that led to the identification of 11 potential candidates [8]Through rigorous analysis over a period of several years, we determined that three candidates (TcG1, TcG2, TcG4) were maximally relevant for vaccine development [9]. These three candidates were phylogenetically conserved in clinically important *T*. *cruzi* strains, expressed in infective and intracellular stages of the parasite [8, 9], and recognized by immunoglobulins and CD8^+^T cells in multiple *T*. *cruzi*-infected hosts [9, 10]. When individually delivered as a DNA-prime/DNA-boost vaccine along with adjuvants (IL-12- and GM-CSF-encoding plasmids) in mice, these antigens elicited a significant trypanolytic antibody and Th1 cytokine (IFN-γ) response, a property that has been associated with immune control of *T*. *cruzi* [8]. Co-delivery of these antigens as DNA vaccine (TcVac1) induced additive immunity and higher degree of protection from *T*. *cruzi* infection than was observed with single vaccine candidates in mice [9]. When tested in dogs, TcVac1 elicited a parasite-specific IgM and IgG (IgG2>IgG1) response but phagocytes’ activity was suppressed resulting in parasites’ escape and dissemination to tissues [10]. Consequently, TcVac1-immunized dogs moderately controlled the chronic parasite persistence and histopathologic cardiac alterations, and remained infective to triatomines [10]. Recent studies have tested several other antigenic candidates as DNA vaccine for their prophylactic and therapeutic efficacy against Chagas disease [11, 12]. Results of these vaccines are encouraging. However, till to date no anti-*T*. *cruzi* vaccine has reached the expected results of producing sterile immunity in dogs.

In this study, we chose to test the protective efficacy of a DNA-prime/inactivated *T*. *rangeli*-boost vaccine (TcVac4) against *T*. *cruzi* infection and Chagas disease in dog model. The use of heterologous DNA-prime/inactivated microorganism-boost vaccine [13] or inactivated microorganism-prime/DNA-boost vaccine [14] has been previously reported with promising results. We included inactivated *T*. *rangeli* as a booster vaccine dose for several reasons: One, *T*. *cruzi* lysates have been previously tested and shown to provide limited or no protection. Though reason for inefficacy of a *T*. *cruzi* epimastigote-based vaccine is not known, it is likely that diversity in the protein expression pattern in epimastigote versus infective/intracellular stages of *T*. *cruzi* and the presence of large family of proteins (e.g. trans-sialidase and mucins) may result in a lack of protective immunity. Two, *T*. *rangeli* exhibits significant homology (>60%) with *T*. *cruzi* proteome [15, 16] but is non-pathogenic for mammals [17, 18] and, thus, require no specific biosafety lab facility for culturing in large batches. Three, mice immunized with glutaraldehyde-fixed *T*. *rangeli* elicited B and T responses that recognized *T*. *cruzi* antigens [19, 20]. Consequently, *T*. *rangeli*-immunized mice were equipped to control challenge infection with *T*. *cruzi* evidenced by a significant reduction in mortality and parasitemia, and absence of histopathological lesions [19, 20]. *T*. *rangeli* based vaccine was also tested in dogs with positive results; dogs immunized with glutaraldehyde-inactivated *T*. *rangeli* epimastigotes exhibited reduced parasitemia after challenge infection with *T*. *cruzi*, and subsequently were less infective to triatomines as compared to controls [21]. We discuss the protective immunity to TcVac4 in dogs and potential utility of this vaccine composition in controlling parasite dissemination in the domestic cycle of transmission.

## Materials and Methods

### Animals and parasite

Twenty-one mongrel dogs (10 males and 11 female, 3–4 months old) were acquired locally and kept in the animal facility at the UAEM Research Center. Animals were included in the experiment when they were > 8-months old (weight: 8–12 kg). All dogs were confirmed to be free of cardiac abnormalities by electrocardiography (EKG) and free of *T*. *cruzi* infection by microscopic examination of blood smears and serological evaluation of anti-*T*. *cruzi* antibodies using an enzyme-linked immunosorbent assay (ELISA) [10]. During the adaptation period, dogs were vaccinated against the regional infectious diseases (Canine distemper, Parvovirus infection, Canine hepatitis, Leptospirosis, and Rabies) and treated against worms. Animals received commercial dog food, according to their physiologic development and water *ad libitum*. All experimental protocols were conducted under the technical specifications for the production, care and use of laboratory animals from the Norma Oficial Mexicana (NOM-62-ZOO-1999) [22], and the council for international Organizations of Medical Science. Dogs were sedated and euthanized according to the Norma Oficial Mexicana (NOM-033-Z00-1995) [23]. All protocols were approved by the Laboratory Animal Care Committee at the Facultad de Medicina Veterinaria y Zootecnia of the Universidad Nacional Autónoma de México (UNAM). Trypomastigotes of *T*. *cruzi* (*Sylvio*X10/4) were maintained and propagated by continuous *in vitro* passage in C2C12 cells.

### Vaccine

Animals were immunized with DNA-prime/inactivated *T*. *rangeli*-boost (TcVac4) vaccine. For the DNA vaccine, cDNAs for TcG1, TcG2 and TcG4 (*Sylvio*X10/4 isolate, Genbank: AY727914, AY727915 and AY727917, respectively) were cloned in the eukaryotic expression plasmid pcDNA3.1 [8]. Plasmids encoding canine interleukin (IL)-12 (p40 and p35 subunits fused to express heterodimeric protein) and canine granulocyte- macrophage colony stimulating factor (GM-CSF) have been previously described [10]. Recombinant plasmids were transformed into *E*. *coli* DH5-alpha-competent cells, grown in LB-broth containing 100-μg/ml ampicillin, and purified by anion exchange chromatography using the Qiagen maxi prep kit (Qiagen, Chatsworth, CA). For booster doses, epimastigotes of *T*. *rangeli* (Guatemala strain, a kind gift from Dr. Jorge Ricardez Esquinca at Facultad de Medicina Humana, Universidad Autónoma de Chiapas, México) were maintained in axenic culture and propagated in LIT media [24]. *T*. *rangeli* epimastigotes (1x10^9^/ml) were inactivated with 0.1% glutaraldehyde solution (Sigma-Aldrich), washed thrice with PBS, and emulsified in PBS containing 500-μg/mL saponin adjuvant (Sigma-Aldrich) [21].

### Immunization and challenge infection

To evaluate the prophylactic efficacy of TcVac4 against acute *T*. *cruzi* infection and Chagas disease, we randomly assigned dogs to the following groups: a) pcDNA3.1/no *Tc* (empty plasmid DNA and no challenge infection, n = 6); b) pcDNA3.1/*Tc* (empty plasmid followed by *Tc* challenge infection, n = 6); c) TcVac4/*Tc* (two doses of DNA vaccine followed by two doses of TrIE and challenge infection, n = 6); and d) TrIE/*Tc* (two doses of TrIE followed by challenge infection, n = 3). Each dose of DNA vaccine was delivered at two sites; intramuscular (180 μg each DNA in 0.9 ml PBS) and intradermal (20 μg each DNA in 0.1 ml PBS). TrIE vaccine was also delivered at two sites; subcutaneous (0.9x10^9^ TrIE in 0.9 ml PBS-saponin) and intradermal (1x10^8^ TrIE in 0.1 ml PBS- saponin).

All vaccine doses were given at 15 days interval, and challenge infection with *T*. *cruzi* (3.5x10^3^ culture-derived trypomastigotes/kg body weight, intraperitoneally) was performed six-weeks after the last vaccine dose. The selected dose of the parasites was sufficient to produce acute parasitemia within 1–2 weeks of inoculation, and electrocardiographic changes within 6–8 weeks post-infection [10, 25]. All dogs were observed daily for general physical condition, at weekly intervals for clinical condition, and at 2-week intervals for cardiac function.

### Parasitological assessment

Blood samples were collected beginning day 5 pi, on alternate days up to 50 days pi; and at two-week intervals thereafter. Parasitemia was measured using hemocytometer counts of 5 μl blood mixed with equal volume of ACK red blood cell lysis buffer. Xenodiagnostic analysis was performed as previously described [10]. Briefly, stage 4 naive triatomines (*M*. *longipennis*) were fed on dogs (6 nymphs per dog) from all treatment groups on days 35 pi. Fecal samples from triatomines were collected at day 60 after feeding, and analyzed by light microscopy to detect epimastigote and/or metacyclic trypomastigotes. One hundred microscopic fields were analyzed for each fecal sample, and triatomines were considered *T*. *cruzi* positive when at least one parasite was detected.

### Serology

Sera samples were obtained before immunization and at two-week intervals after each immunization and challenge infection. Flat bottom 96-well plates (High binding, Costar) were coated overnight with soluble fraction of *T*. *cruzi Sylvio*X10/4 trypomastigotes lysate (5-μg protein/100-μl/well, diluted in NaHCO_3_ solution, pH 9.6). Plates were washed, and then incubated at 37°C for 2 h each with sera samples (1:100–1:1000 dilution, 100-μl/well) and horseradish peroxidase-conjugated sheep polyclonal-anti-dog IgG, goat polyclonal-anti-dog IgG1 or sheep polyclonal-anti-dog IgG2 antibody (1:1000 dilution, 100-μl/well). All antibodies were purchased from Bethyl Laboratories, and diluted in PBS-0.1% Tween-20 (PBS) containing 0.5% NFDM. Color was developed by incubation with 100-μl/well Sure Blue TMB substrate (Kirkegaard & Perry Labs) at room temperature for 10 min, reaction was stopped with 2N sulfuric acid, and change in color monitored at 450 nm using an Epoch microplate reader and Gene 5 (v.2.0) software (Biotek, VT, USA). Sera samples from chronically-infected dogs with confirmed *T*. *cruzi* infection and from healthy domestic dogs were used as positive and negative controls, respectively. The cut off value for ELISA was established as mean O.D. at 450 nm from negative controls ± 2 S.D. [10].

### Electrocardiography

Cardiac parameters were monitored for all dogs before they were included in the study, and then after challenge infection, at 2-week intervals up to 8-weeks and at monthly intervals thereafter. We used electrocardiograph (Stylus, EK-8, USA) setting at 120 V, 60 Hertz, 20 amps, and 25 Watt in all experiments. Six leads (I, II, III, aVr, aVL and aVF) of the electrocardiogram were considered at 1-mV/cm, and a 25 mm/sec paper speed [10]|.

### Necropsy and histological studies

Necropsy was performed on the day animals died due to infection or after humanitarian sacrifice at day 60 pi corresponding to acute infection phase and at day 365 pi corresponding to chronic phase of disease development. Postmortem studies were conducted using standard protocols with emphasis on macroscopic findings related to Chagas disease in heart tissue. Also, liver and spleen were carefully inspected [10, 25].

For histological analysis, tissue samples were fixed in 10% buffered formalin for 24 h, dehydrated in absolute ethanol, cleared in xylene, and embedded in paraffin. Tissue sections (5-μm thick) were stained with hematoxylin and eosin, and evaluated by light microscopy (magnification: 100x and 400x) [10, 25].

### Identification of parasite presence in heart tissue

Tissue parasites were identified by nested PCR. Briefly, total DNA was isolated from cardiac tissue sections using Wizard SV genomic DNA purification System Kit (Promega) following the manufacturer’s instructions. For first PCR amplification, amplicon (350 bp) was obtained from the conserved region of the mini exon sequence (GenBank accession # X62674) [26]. The reaction mixture consisted of Taq buffer (750 mM Tris-HCL, pH 8.8, 200 mM NH_4_SO4 and 0.1% Tween 20), 0.2 mM dNTPs, 3 mM MgCl2, and 1.25 U/50 μl Taq polymerase (Go Taq Flexi DNA Polymerase). The reaction was started with addition of 1 μM of each oligonucleotide (TC2F: 5′-CCTGCAGGCACACGTGTGTGTG-3′ and TCR: 5′-CCCCCCTCCCAGGCCACACTG-3′) and 0.1 μg DNA template; and PCR was performed as follows: initial denaturation at 94°C/4 min, followed by 30 cycles of denaturation (94°C/1 min), annealing (55°C/30 sec) and extension (72°C/1 min), and a final incubation at 10°C/5 min.

For the second round of nested PCR, internal primers (Tc2F: 5′-GCACGGTGTTCTGTCTTGTC-3′ and Tc2R: 5′-ATCAGCGCCACAGAAAGTGT-3′), designed using the Primer3plus software [27] were included in the reaction to amplify a 197 bp band. The reaction was initiated using 1 μl of the amplicon from the 1^st^ PCR reaction as template DNA, and amplification for nested PCR was performed as follows: initial denaturation 94°C/4 minutes, followed by 30 cycles of denaturation (95°C/30 sec), annealing (51°C/30 sec) and extension (75°C/1 min), and a final incubation at 10°C/5 minutes. Amplicons were resolved by agarose gel (3%) electrophoresis, and gels were stained with ethidium bromide (0.5 μg/mL), and visualized and imaged using a UV transilluminator mounted with a digital Kodak DC120 camera.

### Statistical analysis

Serological and parasitemia data were analyzed in duplicate and expressed as mean ± SD. Data were assessed for normal distribution by Kolmogorov-Smirnov (K-S) test [28] and checked by histograms and Q-Q plots. Data did not fit normal distribution, and therefore, were analyzed by a 1-way analysis of variance (ANOVA). The mean differences were determined by Tukey’s post-hoc test (comparison of multiple groups). Statistical analysis was performed using Statistical Analysis Systems (SAS) Software version 9.0 [29]. Differences were considered significant at p<0.05.

## Results

### Clinical and electrocardiographic findings in chagasic dogs vaccinated with TcVac4

Electrocardiographic findings were graded according to the severity of the abnormalities from 0 (normal) to 10 (most severely affected) [10]. We noted no apparent clinical signs of cardiac dysfunction in dogs during routine evaluation in the adaptation period or during the immunization period. After challenge infection with *T*. *cruzi*, all dogs, except TcVac4-vaccinated and negative control (pcDNA3.1/no *Tc*) dogs, displayed a degree of electrocardiographic alterations during 30–60 days pi, corresponding to the acute infection phase (Table 1). Up to 67% of the pcDNA3.1/*Tc* dogs exhibited severe EKG abnormalities (average grade 6.8) in response to *T*. *cruzi* infection, and were diagnosed with one or more of the following cardiac abnormalities: deviation of the electrical axel, low voltage complexes, and inter-ventricular conduction problems. TrIE/*Tc* dogs exhibited moderate level of cardiac dysfunction (average grade 2.6), and displayed repolarization problems and low voltage complexes. In comparison, TcVac4/*Tc* dogs exhibited no *T*. *cruzi*-induced changes in cardiac hemodynamics and their EKG profile was similar to that noted in pcDNA3.1/no *Tc* mice (Table 1).

**Table 1:** Electrocardiographic evaluation of TcVac4-immunized dogs post challenge infection with *T*. *cruzi*.

Dogs per group	pcDNA3.1/no *Tc*	pcDNA3.1/*Tc*	TcVac4/*Tc*	TrIE/*Tc*
**Day 60 post-infection**				
Dog 1	0	10	0	0
Dog 2	0	3	0	8
Dog 3	0	9	0	0
Dog 4	0	9	0	ND
Dog 5	0	9	0	ND
Dog 6	0	1	0	ND
Abnormalities	None	HAD, LVC, ICP	None	RP, LVC
**Total**	0/6 (0%)	4/6 (67%)	0/6 (0%)	1/3 (33%)
**Day 365 post-infection**				
Dog 4	0	9	0	ND
Dog 5	0	9	8	ND
Dog 6	0	1	0	ND
Abnormalities	None	AVB, ADR	Small QRS	ND
**Total**	0/6 (0%)	2/3 (66%)	1/3 (33%)	ND

Dogs were vaccinated and challenged with *T*. *cruzi* as described in Materials and Methods. Cardiac hemodynamics were monitored by electrocardiography, and graded as 0–10, 10 being most severe. HAD, High axis deviation; LVC, Low Voltage Complex; ICP, Interventricular conduction Problems; RP, Repolarization Problems. Type II (Mobitz) AVB, 2^nd^ degree Atrio-Ventricular Block; ADR, Axis deviation to the right; Small QRS, reduced QRS wave; ND, Not determined (animals not included for the study of these parameters).

During the chronic phase of disease development (365 days pi), all dogs from the pcDNA3.1/*Tc* group exhibited Chagas disease associated cardiac abnormalities evidenced by deviation of axel to the right, 2° degree type II Mobitz block, and low voltage complexes (average grade 6.3). In comparison, TcVac4/*Tc* dogs presented normal electrocardiograms, and only one dog in this group developed mild electrocardiographic alterations with low voltage R complex (average grade 2.6) (Table 1). TrIE/*Tc* dogs were not followed in the chronic phase of infection. These data suggest that TcVac4 vaccinate was efficacious in preserving the cardiac function that otherwise was severely compromised in response to *T*. *cruzi* infection and Chagas disease development.

### TcVac4 induced antibodies: expansion in response to challenge infection

**Fig 1:**
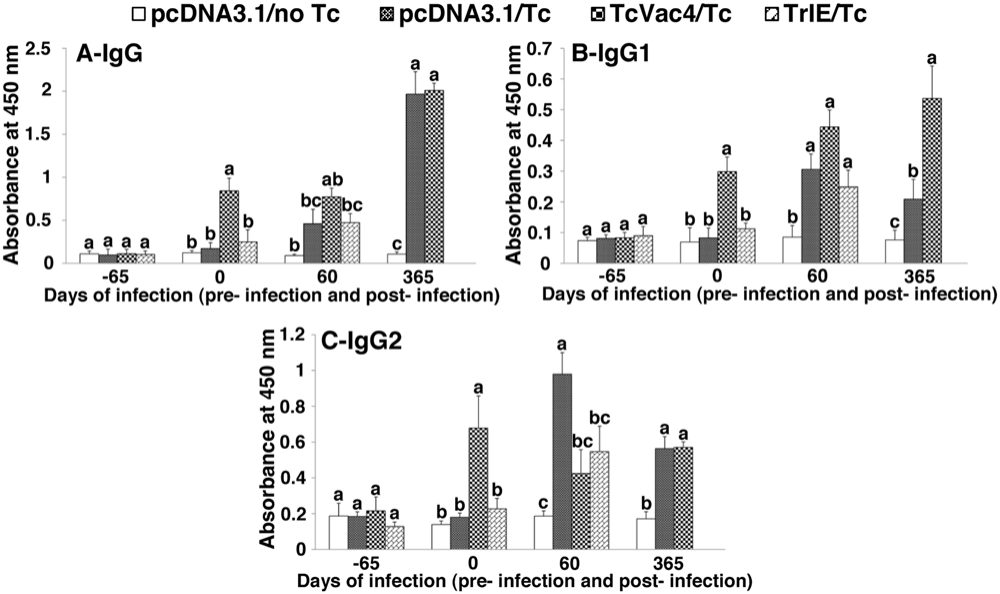
TcVac4-induced antibody response in dogs (± *T*. *cruzi*). Dogs were vaccinated with TcVac4 or TrIE only and infected with *T*. *cruzi*, as described in Materials and Methods. Shown are sera levels of *T*. *cruzi*-specific IgG **(A)**, IgG1 **(B)**, and IgG2 **(C)** antibody subtypes, determined by an ELISA. Dogs given pcDNA3.1/no infection and dogs given pcDNA3.1/*T*. *cruzi* were included as negative and positive controls, respectively. The serology time points are described as day -65 = basal response before immunization, day 0 representing antibody response after last immunization but before challenge infection, day 60 post challenge equivalent to acute infection phase, and day 365 post challenge equivalent to chronic disease phase. Each bar represents the absorbance mean value ± standard deviation. Within the same time point, statistical differences (p < 0.05) among groups are shown with different characters above the bars according to Tukey’s test.

Sera levels of *T*. *cruzi*-specific antibodies were determined by an ELISA test. All dogs were seronegative before vaccination was initiated. *T*. *cruzi*-specific antibody response (IgGs, 1:100-dilution) was detectable after first vaccine dose, and gradually increased with subsequent doses. The antibody response examined after last vaccine dose is shown in Fig 1. TcVac4/*Tc* dogs exhibited 2.2–4.9-fold higher level of *T*. *cruzi*-specific IgGs as compared to that noted in TrIE/*Tc* dogs (Fig 1A, p<0.05). Likewise, TcVac4/*Tc* dogs exhibited >3-fold increase in IgG1 and IgG2 levels (IgG2/IgG1 ratio: 2.23) when compared to that noted in sera of TrIE/*Tc* dogs (Fig 1B and 1C, p<0.05).

All animals, irrespective of vaccination status, responded to challenge infection by a significant expansion of antibody response. During the acute infection phase, the IgG and IgG1 levels were increased by 3.5 fold, and a trend was observed as following: TcVac4 > TrIE = pcDNA3.1 only (Fig 1A and 1B). The IgG2 levels were increased by >5-fold in pcDNA3.1/*Tc* dogs while TcVac4/*Tc* and TrIE/*Tc* dogs exhibited 2-3-fold increase in IgG2 levels when compared to normal controls (Fig 1C). The IgG2/IgG1 ratios were 3.2, 0.95 and 2.2 in acutely infected dogs immunized with pcDNA3.1, TcVac4, and TrIE, respectively (Fig 1B and 1C).

At 360 days pi corresponding to chronic phase of disease development, the IgG (>10-fold) and IgG2 (>3-fold) responses remained substantially high in TcVac4/*T* dogs as well as in pcDNA3.1/*Tc* dogs (Fig 1A and 1C). TcVac4/*Tc* dogs also maintained a high level of IgG1 response resulting in a balanced IgG subtypes (IgG1 = IgG2) while chronically infected control dogs (pcDNA3.1/*Tc*) exhibited a significantly lower level of IgG1 subtype (IgG2/IgG1 = 3.76) (Fig 1B and 1C). Together, the results presented in Fig 1 suggested that TcVac4 elicited a high level of *T*. *cruzi* specific antibody response (IgG2>IgG1) that was expanded in response to challenge infection and maintained during chronic phase with a balanced level of IgG1 and IgG2 subtypes. The TrIE-induced antibody response was not associated with protection from chronic disease.

### TcVac4-induced control of blood parasitemia, parasite transmission to triatomines, and tissue parasite burden

**Fig 2:**
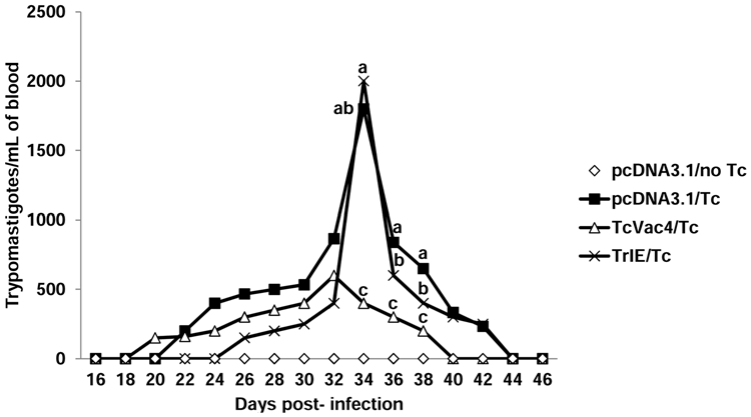
Blood parasitemia control in dogs immunized with TcVac4. Dogs were immunized with TcVac4 or TrIE, and infected with *T*. *cruzi* as in Fig 1. Blood samples were evaluated for parasitemia by light microscopy at alternate days post-infection. Shown are data from day 16 to 46 after challenge infection, presented as mean values (n = 6/group). Different letters above lines show statistical differences (p< 0.05) among treatments within the same day of sampling according to Tukey’s test.

Parasitemia was detected in all experimentally infected dogs. In general, pre-patent period lasted until 16 days pi and peak parasitemia was reached between days 32 and 37 pi in all infected dogs (Fig 2). The TcVac4/*Tc* dogs exhibited 4-fold lower level of peak parasitemia than was noted in TrIE/*Tc* or pcDNA3.1/*Tc* dogs. Further, in TcVac4/*Tc* dogs, parasitemia became undetectable at day 40 pi that was four days earlier than was noted in animals from other infected groups (Fig 2).

Xenodiagnostic studies were performed to assess if TcVac4 vaccine altered the infectivity of dogs to triatomines. For this, triatomines were fed on dogs during the acute infection phase when peak parasitemia was observed (i.e., day 36 pi), and feces were analyzed at day 60 post-feeding for the detection of parasites by light microscopy. Interestingly, triatomine survival frequency was affected by the infection status of the host evidenced by 100%, 72%, 83% and 72% survival rate of insects fed on pcDNA3.1/no *Tc*, pcDNA3.1/*Tc*, TcVac4/*Tc*, and TrIE/*Tc* dogs, respectively (Table 2). Considering only the surviving bugs, the rate of infection (i.e. positive for parasite detection) was observed to be 0%, 89%, 50% and 69% of triatomines fed on pcDNA3.1/no *Tc*, pcDNA3.1/*Tc*, TcVac4/*Tc* and TrIE/*Tc* dogs, respectively (Table 2). Together the results presented in Fig 2 and Table 2 suggested that neither of the vaccine compositions were effective in preventing infection or early rise in acute parasitemia. However, TcVac4 was most effective in reducing the peak parasitemia, time-course of parasitemia, and dogs’ infectivity to triatomines.

**Table 2:** Frequency of parasite transmission from TcVac4-vaccinated dogs post challenge infection with *T*. *cruzi*.

Groups	No. of surviving bugs post-feeding/Total bugs (%)	No. of infected bugs/surviving bugs post-feeding (%)
pcDNA3.1/no *Tc* (n = 6)	36/36 (100%)	0/36 (0%)
pcDNA3.1/*Tc* (n = 6)	26/36 (72%)	23/26 (83%)
TcVac4/*Tc* (n = 6)	30/36 (83%)	15/30 (50%)
TrIE/*Tc* (n = 3)	13/18 (72%)	9/13 (69%)

Dogs were vaccinated and challenged with *T*. *cruzi* as described in Materials and Methods. Triatomines (*Meccus longipennis*) were fed on dogs at day 36 pi and infection of insects was evaluated 60 days post-feeding.

Dogs in all groups, before or after challenge infection, presented no apparent signs of clinical illness during the routine physical exam. All dogs in TcVac4/*Tc* and pcDNA3.1/*Tc* groups survived well through the acute phase of infection. On day 60 pi, three dogs from these groups were harvested for anatomical and pathological evaluation and remaining animals in these groups survived till day 365 pi when the experiments were terminated. One dog in TrIE/*Tc* group succumbed to challenge infection on day 40 pi, and other 2 dogs in this group were harvested at day 60 pi for pathologic evaluations.

Since amastigote nests are normally not observed during the chronic phase of infection by histological techniques and end-point PCR is not highly sensitive in detecting low levels of tissue parasite burden, we performed nested PCR to evaluate tissue-parasite burden in chronically-infected dogs. Diagnostics of parasite persistence in cardiac tissue by a nested PCR demonstrated that all infected animals were positive for parasite DNA. These data, along with those presented in Fig 2 and Table 2, suggested that immunization with TcVac4 was effective in reducing the acute parasitemia and parasite transmission to triatomines; however, parasite persistence in tissues was not abrogated by TcVac4 vaccine.

### Necropsy and histological studies in acutely- and chronically-infected dogs (± TcVac4)

**Fig 3:**
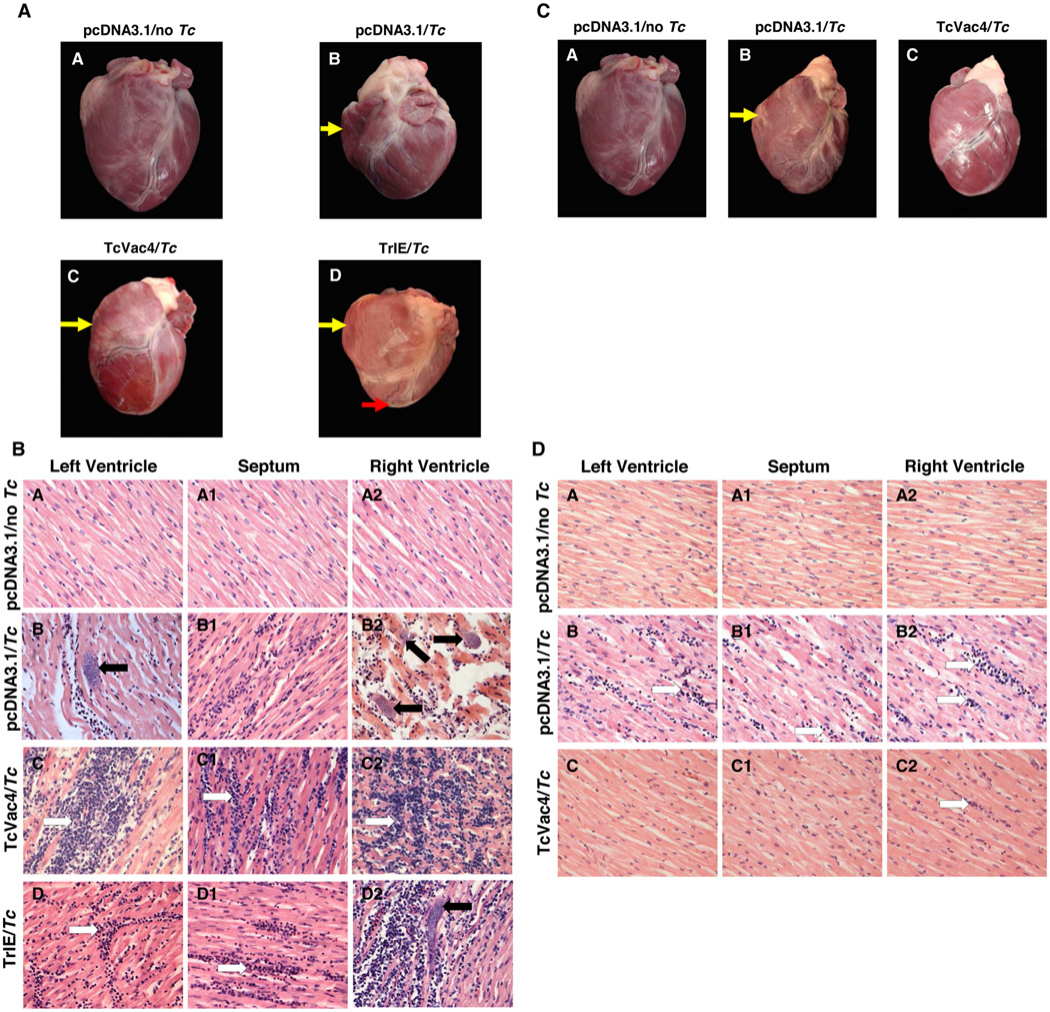
Macroscopic and microscopic alterations in the heart of *T*. *cruzi*-infected dogs (±TcVac4). **(A)** Morphologic alterations of heart in acute infection phase (±TcVac4). Shown are representative heart pictures from dogs included in pcDNA3.1/no *Tc* (A), pcDNA3.1/*Tc* (B), TcVac4/*Tc* (C) and TrIE/*Tc* (D) groups, harvested at day 60 pi. Yellow arrows indicate right ventricular dilation. Red arrows indicate the pale/striated areas of fibrotic tissue. **(B)** Histological analysis of heart tissue-sections in acutely-infected dogs (±TcVac4). Heart tissue sections (5-μM) from left ventricle, septum, and right ventricle were obtained at 60 days pi, and stained with hematoxylin-eosin. Shown are representative micrographs of heart from dogs injected with pcDNA3.1/*Tc* (B, B1, B2), TcVac4/*Tc* (C, C1, C2), and TrIE/*Tc* (D, D1, D2). Micrographs from pcDNA3.1/no *Tc* (A, A1, A2) are shown as negative controls. Black arrows show amastigotes nests. White arrows show inflammatory infiltrate in the myocardium. **(C)** Morphologic alterations of heart in chronic disease phase (±TcVac4). Shown are representative heart pictures from dogs included in pcDNA3.1/no *Tc* (A), pcDNA3.1/*Tc* (B), TcVac4/*Tc* (C) and TrIE/*Tc* (D) groups, harvested at day 365 pi. Black arrows point out the right ventricular dilation. **(D)** Histological analysis of heart tissue-sections in chronically-infected dogs (±TcVac4). Heart tissue sections (5-μm) from left ventricle, septum, and right ventricle were obtained at 365 days pi, and stained with hematoxylin-eosin. Shown are representative micrographs of chronically-infected dogs injected with pcDNA3.1 only (B, B1, B2) or immunized with TcVac4 (C, C1, C2). Micrographs from pcDNA3.1/no *Tc*, i.e. normal/healthy controls (A, A1, A2) are shown for comparison. White arrows mark infiltrating inflammatory cells in myocardium.

Anatomopathological analysis of the heart, performed at 60 days pi (acute infection phase), showed biventricular dilated cardiomyopathy and focal hemorrhages and pale zones indicative of tissue damage and fibrosis in both ventricles. Animals from all infected groups displayed similar type of pathologies irrespective of the vaccination status. However, heart lesions in response to acute infection were milder in TcVac4/*Tc* dogs than those noted in pcDNA3.1/*Tc* or TrIE/*Tc* dogs (Fig 3A and Table 3). Further, moderate splenomegaly and hepatomegaly, associated with pale zones resembling infarct areas were presented in acutely-infected pcDNA3.1/*Tc* or TrIE/*Tc* dogs. TcVac4/*Tc* dogs exhibited severe splenomegaly indicative of strong immune response but only mild lesions in the liver (Table 3). Histological studies validated the macroscopic observations at day 60 pi. We observed severe focal myocardial lymphoplasmacytic inflammation in TcVac4/*Tc* dogs, while moderate diffused myocardial lymphoplasmacytic inflammation was recorded in the myocardium of pcDNA3.1/*Tc* and TrIE/*Tc* dogs during the acute infection phase (Fig 3B). The extent of infiltration of lymphocytes and polymorphonuclear leucocytes in the heart in acute infection phase was maximally noted in TcVac4/*Tc* dogs followed by TrIE/*Tc* and pcDNA3.1/*Tc* dogs. Likewise, cardiomyocyte necrosis was observed more frequently in TcVac4/*Tc* and TrIE/*Tc* dogs than in pcDNA3.1/*Tc* dogs that presented a moderate amount of necrotic cells. In contrast, amastigote nests associated with acute infection were abundant (3–6 foci of pseudocysts/microscope field) in cardiac tissue of pcDNA3.1/*Tc* and TrIE/*Tc* dogs, and scarce (0–1 foci of pseudocysts/mf) in TcVac4/*Tc* dogs (Fig 3B and Table 3). These data suggested that immunization with TcVac4 vaccine resulted in an immune response associated with extensive inflammatory infiltrate in the heart upon challenge infection; and subsequently tissue parasite burden was controlled. In comparison, TrIE vaccine composition was not effective in controlling the acute tissue parasite burden.

**Table 3:** Anatomopathological and histopathological abnormalities in dogs during the acute and chronic phases of *T*. *cruzi* infection and disease development (± TcVac4 vaccine).

	pcDNA3.1/no *Tc*	pcDNA3.1/*Tc*	TcVac4/*Tc*	TrIE/*Tc*
**Macro-anatomopathology (day 60 post-infection)**				
LV dilatation	-	+++	++	+++
RV dilatation	-	+++	++	++
Pale striated epi/myocardium	-	+	++	+++
Splenomegaly	-	++	+++	+++
Hepatomegaly	-	++	+	++
**Myocardial histopathology (day 60 post-infection)**				
Focal lympho-plasmacytes	-	++	+++	+++
Diffused lympho-plasmacytes	-	++	++	++
Polymorphonuclear leukocyte/lymphocyte	-	+	+++	++
Necrosis of cardiomyocytes	-	++	+++	+++
Amastigotes nests	-	++	+	+++
**Macro-anatomopathology (day 365 post-infection)**				
LV dilatation	-	+++	+	ND
RV dilatation	-	+++	++	ND
Splenomegaly	-	+	+	ND
Heptomegaly	-	+	+	ND
**Myocardial histopathology (day 365 post-infection)**				
Focal lympho-plasmacytes	-	+	+	ND
Diffused lympho-plasmacytes	-	++	+	ND
polymorphonuclear leukocyte/lymphocyte	-	+++	+	ND	
Necrosis of cardiomyocytes	-	+	+	ND

Anatomopathological and histopathological evaluations were conducted during acute (day 60 pi) and chronic (day 365 pi) phases of infection and disease development. Classification of abnormalities was conducted according to [25] and [39], and represented as-, None; +, slight; ++, moderate; and +++, severe; ND, Not determined (animals not included for the study of these parameters).

At one-year post-challenge infection, mild splenomegaly and hepatomegaly were present in all chronically-infected dogs (Table 3). The lesions presented in the heart of chronically infected pcDNA3.1/*Tc* dogs were more severe than that noted in TcVac4/*Tc* dogs (Fig 3C and Table 3). Histological studies showed the microscopic lesions constituted of focal myocardial lymphoplasmacytic inflammation and necrotic cardiomyocytes in chronically infected dogs (Fig 3D). The pcDNA3.1/*Tc* dogs exhibited severe damage, i.e., moderate diffused myocardial lymphoplasmacytic inflammation and severe infiltration of lymphocytes and polymorphonuclear leukocytes (Fig 3). These lesions were fewer and milder in chronically infected TcVac4/*Tc* dogs (Fig 3D and Table 3). Together, these data suggested that the TcVac4-vaccinated dogs were better equipped in controlling the chronic parasite persistence and pathological inflammation in the heart as well as the associated pathology in other organs that was otherwise evident in chronically-infected control dogs.

## Discussion

In this study, we have tested the efficacy of a DNA-prime/TrIE-boost (TcVac4) vaccine in dogs. The candidate antigens TcG1, TcG2, and TcG4 used as DNA vaccine in the study are conserved in multiple, clinically-relevant isolates of *T*. *cruzi*, expressed in infective and intracellular stages of *T*. *cruzi*, recognized by antibody and T cell immunity in infected mice [8, 30], and recently shown to be serologically reactive in infected humans [31]. IL-12 and GM-CSF expression plasmids were used as adjuvants because these cytokines induce antigen presentation and B and T cell responses [32].

Our previous experience with testing the efficacy of a multi-component DNA-prime/DNA-boost vaccine (TcVac1) constituted of TcG1, TcG2, and TcG4 in dogs was moderately encouraging [10]. Dogs vaccinated with TcVac1 elicited antigen-specific IgM and IgG (IgG2>IgG1) antibodies, and upon challenge infection, responded by a rapid expansion of antibodies but a moderate level of CD8^+^ T cell proliferation and IFN-γ production, and suppression of phagocytes’ activity. Subsequently, TcVac1 provided an early control of acute parasitemia but no significant benefits in controlling the myocardial parasite burden, electrocardiographic abnormalities or histopathologic cardiac alterations, the hallmarks of acute Chagas disease [10]. These data indicated that, even though TcVac1 primed an initial immune response against *T*. *cruzi* infection, the vaccine needed improvements. We chose to strengthen the TcVac1 efficacy with heterologous booster dose constituted of *T*. *rangeli* inactivated epimastigotes. Our decision was based on the fact that *T*. *rangeli* is nonpathogenic to humans [18], exhibits ~60% cross-reactive antigenicity with *T*. *cruzi* [17], and immunization with *T*. *rangeli*-based vaccines haven been shown to elicit partial protection against experimental *T*. *cruzi* infection in dogs and mice [19–21, 33, 34]. *T*. *rangeli*-based protection from *T*. *cruzi* was associated with potent parasite-specific antibodies and elevated serum levels of proinflammatory cytokines (IL-12, IFN-γ > IL-6, TNF-α > IL-10) that subsequently resulted in absence of histopathological lesions in mice [19]. Others have shown the DNA prime/inactivated microorganism vaccine approach was significantly better than the individual components in providing protection against *Mycobacterium tuberculosis* in mice [14] and rabies [13]. Importantly, we have noted that subunit vaccine delivered by heterologous DNA-prime/protein-boost approach [30] was more efficacious in eliciting potent immunity to *T*. *cruzi* infection than was observed with DNA-prime/DNA-boost homologous vaccine in mice [9].

Herein, we report that immunization with TcVac4, delivered as two doses of subunit DNA vaccine and two doses of TrIE, elicited a strong parasite-specific IgG response with predominance of IgG2 subtype (IgG2/IgG1 = 2.7). Upon challenge infection, IgG1 response was expanded and IgG2 response barely changed, resulting in a balanced IgG2/IgG1 ratio (0.95) in TcVac4/*Tc* dogs. A strong and balanced antibody response (IgG1 = IgG2) was maintained in TcVac4 vaccinated dogs during the chronic phase (365 days pi), while dogs from pcDNA3.1/*Tc* group exhibited an IgG2 biased response (IgG2/IgG1 = 3.76). DNA vaccines have previously been shown to trigger IgG2 biased antibody response [6, 10, 11, 35]. In dogs, IgG2 antibodies are known to support Fc region mediated effector functions, such as antibody-dependent cellular cytotoxicity and complement-dependent cytotoxicity [36]]. Yet, our results suggest that quality of the IgG2 antibodies and a strong level of IgG1 antibodies are more important than the mere IgG2-biased response in controlling infection. This notion is supported by the observation that TcVac4-induced balanced antibody responses were effective in controlling blood parasitemia, tissue parasite replication, and parasite dissemination to triatomines, while a highly IgG2 biased antibody response in pcDNA3.1/*Tc* and TrIE/*Tc* dogs provided no protection from acute infection [10, 11]. Guedes et al. [37] have reported that a strong IgG2-biased antibody response was associated with cardiomegaly in 50% of beagle dogs chronically infected with *T*. *cruzi* Berenice-78 strain and 100% of dogs infected with Y or ABC *T*. *cruzi* strains, thus suggesting that a strong IgG2 response (without IgG1 antibodies) caused more cardiac damage than control of infection.

TcVac4 had a dramatic effect on parasitemia levels during the acute phase of infection with 500 parasites/mL blood, in comparison with 2000 parasites/mL found in control animals; additionally the duration of the parasitemia was also reduced in TcVac4/*Tc* dogs. Others have also reported a degree of parasitemia control by DNA or protein vaccine in dogs. For example, Rodriguez-Morales et al. [6] and Arce-Fonseca et al. [11] using TcSSP4- and TcSP-encoding DNA vaccine, and Quijano–Hernandez et al. [12] using *TSA-1/Tc24*-encoding DNA vaccine, have found a reduction in acute parasitemia and the duration of parasitemia in vaccinated dogs. Also in a previous study, using the TcVac1 multicomponent vaccine, we found a reduction in duration of microscopically detectable parasitemia although a reduction in number of circulating parasites/mL was not observed during the acute phase of infection [10]. Although all these vaccines, including TcVac4, have provided some protection in reducing parasitemia during the acute phase of the infection, future vaccine improvements will be necessary as sterile immunity was not achieved.

We found that electrocardiographic abnormalities associated with acute parasite burden were prevented in TcVac4/*Tc* dogs, evidenced by normal electrocardiographic readings in all animals in this group. During the chronic phase, TcVac4/*Tc* dogs continued to exhibit a normal electrocardiogram, and only one dog in this group developed aberrant QRS wave. The pcDNA3.1/*Tc* dogs displayed severe cardiac abnormalities, such as high axis deviation, interventricular conduction problems, and low voltage complex in response to acute infection, and these abnormalities persisted during the chronic disease phase when dogs also exhibited other EKG problems, such as axis deviation to the right; AVB, Type II (Mobitz) 2^nd^ degree atrio-ventricular block (Table 1). This information indicates that even if damage was not completely controlled, TcVac4/*Tc* animals were better equipped than the pcDNA3.1/*Tc* in preventing the chronic infection associated cardiac dysfunction. Reduction in abnormal EKG readings have also been reported by the use of *TSA-1/Tc24* DNA vaccine [12] wherein authors reported that 33% of vaccinated/infected animals and 71% of the non-vaccinated/infected dogs had EKG alterations. Rodriguez-Morales et al. [35] have shown vaccination with TcSSP4 provided complete control of EKG alterations induced by *T*. *cruzi Ninoa* isolate. Although results with TcSSP4 seem more encouraging that the results found with TcVac4, direct comparison of these studies can not be done because *Ninoa*, the strain used by Rodriguez-Morales group, is not as pathogenic to dogs as *Sylvio* X10/4 *T*. *cruzi* strain.

The observation of an intense focal myocardial lymphocytic and polymorphonuclear leukocyte infiltration in acutely-infected TcVac4/*Tc* dogs suggests that TcVac4 also primed cell-mediated immune response that was significantly expanded to control intracellular infection. It is important to note that TcVac4 efficacy was observed against *Sylvio*X10/4 that we have found is very pathogenic in dogs [10, 12]. During the chronic phase, TcVac4/*Tc* dogs exhibited a remarkable reduction in macroscopic lesions as well as inflammatory infiltrate in the myocardial tissue, and these animals mostly had normal cardiac hemodynamics and LV function in comparison with that observed in chronically-infected dogs that were not vaccinated. Similar findings of enhanced inflammatory lesions in acute phase followed by control of chronic inflammation, albeit to a lesser scale, have been reported by others using *TSA-1/Tc24* DNA vaccine [12] and TcSSP4 protein vaccine [35] in dogs.

We also studied the capacity of TcVac4 to block parasite transmission from infected dogs to the vector (*Meccus longipennis*). Our data indicated that triatomines were sensitive to infection with *Sylvio*X10/4 strain of *T*. *cruzi*, because a relatively large proportion of insects fed on infected dogs died few days after feeding, while triatomines fed on naïve dogs had no postprandial mortality (Table 1). Nevertheless, mortality and infection rate of triatomines fed on TcVac4/*Tc* dogs, animals that had shortest peak of parasitemia, was reduced as compared with the triatomines from all other infected groups (Table 2). These data suggest that TcVac4 is not only effective in providing protection to dogs from *T*. *cruzi* infection but is also effective in partially reducing the dogs’ infectivity to triatomines. Our results suggest the vaccination of dogs, via blocking the parasite transmission to triatomines, will potentially be useful in preventing human infection and provide us an impetus to further improve the vaccine efficacy to interrupt the domestic cycle of parasite transmission.

*T*. *rangeli* has previously been reported to induce protection against an experimental infection with *T*. *cruzi* in dogs [21]. Thus, a limited protection afforded by vaccination with TrIE in this study was unexpected. Differences in experimental outcomes between our and previously published literature could probably be explained by methodology dissimilarities. For example, Colombian field isolate of *T*. *rangeli*, used as vaccine by Basso et al. [19, 20] might be from a different lineage than the Guatemalan isolate that we used in this study; and the antigenic differences between the two isolates of *T*. *rangeli* [38] might have influenced their protective capacity when used as vaccine against *T*. *cruzi*. Differences in virulence of Tulahuen isolate used by Basso et al. [21] versus *Sylvio*X10/4 isolate of *T*. *cruzi* used by us might also explain the observed differences in *T*. *rangeli*-based vaccine efficacy. In our experience, *Sylvio*X10/4 is highly pathogenic to dogs [10, 12, 25]. Though the pathogenicity of Tulahuen in dogs was not discussed, the fact that Basso et al. [21] challenged the dogs with 10,000 parasites/kg of body weight, while in the present study 3500 parasites/kg of body weight were used, suggest that Tulahuen is less pathogenic than the *Sylvio*X10/4 in dogs. Moreover, number of booster doses could also have influenced the protective capacity of the vaccine. We immunized dogs with two doses of *T*. *rangeli*-based vaccine while Basso et al [19, 20] immunized dogs thrice. The two-dose TrIE immunization protocol was not sufficient to elicit protective immune response; these animals exhibited low antibody response predominated by IgG2 subtype and only a weak IgG1 antibody response was mounted during immunization as well as post-challenge period. Again, in agreement with Guedes et al [37], a weak IgG1 response observed in TrIE-vaccinated dogs could be related to a poor protection against infection.

In summary, we have shown that TcVac4 vaccine induced a predominant IgG2-biased antibody response that was replaced by a balanced IgG (IgG1 = IgG2) response after challenge infection. TcVac4-vaccinated animals were equipped to reduce parasitemia, heart tissue parasite burden, macroscopic heart damage and electrocardiographic alterations during acute phase of the infection. During the chronic phase, TcVac4-vaccinated animals exhibited few inflammatory foci, no parasite nests and preserved cardiac function. Our data provide impetus to further improve the TcVac4 efficacy and examine its potential utility as a veterinary vaccine.

## References

[1] World Health Organization. Chagas disease: control and elimination. In: Report of the secretariat. WHO, Geneva: UNDP/World Bank/WHO. 2010. Available from http://apps.who.into/gb/ebwha/pdf_files/WHA63/A63_17-en.pdf

[2] Tanowitz HB, Weiss LM, Montgomery SP. Chagas disease has now gone global. PLoS Negl Trop Dis. 2011;5:e1136 10.1371/journal.pntd.0001136 21572510 PMC3082509

[3] Camargo EP. Perspectives of vaccination in Chagas disease revisited. Mem Inst Oswaldo Cruz. 2009;104 Suppl 1:275–280. 19753485 10.1590/s0074-02762009000900036

[4] Docampo R. Recent developments in the chemotherapy of Chagas disease. Curr Pharm Des. 2001;7:1157–1164. 11472259 10.2174/1381612013397546

[5] Coura JR, Borges-Pereira J. Chagas disease. What is known and what should be improved: a systemic review. Rev Soc Bras Med Trop. 2012;45:286–296. 22760123 10.1590/s0037-86822012000300002

[6] Rodriguez-Morales O, Perez-Leyva MM, Ballinas-Verdugo MA, Carrillo-Sanchez SC, Rosales-Encina JL, Alejandre-Aguilar R, et al. Plasmid DNA immunization with *Trypanosoma cruzi* genes induces cardiac and clinical protection against Chagas disease in the canine model. Vet Res. 2012,43:79 10.1186/1297-9716-43-79 23148870 PMC3505182

[7] Vázquez-Chagoyán JC, Gupta S, Jain Garg N. Vaccine Development Against *Trypanosoma cruzi* and Chagas Disease. In: Weiss LM, Tanowitz HB,and Kirchhoff LV, editors. Andvances in Parasitology; 2011 pp. 121–131. 10.1016/B978-0-12-385863-4.00006-X 21820554

[8] Bhatia V, Sinha M, Luxon B, Garg NJ. Utility of *Trypanosoma cruzi* sequence database for the identification of potential vaccine candidates: *In silico* and *In vitro* screening. Infect Immun. 2004;72:6245–6254. 15501750 10.1128/IAI.72.11.6245-6254.2004 PMC523045

[9] Bhatia V, Garg NJ. Previously unrecognized vaccine candidates control *Trypanosoma cruzi* infection and immunopathology in mice. Clin Vaccine Immunol. 2008;15:1158–1164. 10.1128/CVI.00144-08 18550728 PMC2519298

[10] Aparicio-Burgos JE, Ochoa-Garcia L, Zepeda-Escobar JA, Gupta S, Dhiman M, Martinez JS, et al. Testing the efficacy of a multi-component DNA-prime/DNA-boost vaccine against Trypanosoma cruzi infection in dogs. PLoS Negl Trop Dis. 2011;5:e1050 10.1371/journal.pntd.0001050 21625470 PMC3098890

[11] Arce-Fonseca M, Ballinas-Verdugo MA, Zenteno ER, Suarez-Flores D, Carrillo-Sanchez SC, Alejandre-Aguilar R, et al. Specific humoral and cellular immunity induced by *Trypanosoma cruzi* DNA immunization in a canine model. Vet Res. 2013;44:15 10.1186/1297-9716-44-15 23497041 PMC3601012

[12] Quijano-Hernández IA, Castro-Barcena A, Vázquez-Chagoyán JC, Bolio-González ME, Ortega-López J, Dumonteil E. Preventive and therapeutic DNA vaccination partially protect dogs against an infectious challenge with *Trypanosoma cruzi* . Vaccine. 201;31:2246–2252. 10.1016/j.vaccine.2013.03.005 23499599

[13] Biswas S, Reddy GS, Srinivasan VA, Rangarajan PN. Preexposure efficacy of a novel combination DNA and inactivated rabies virus vaccine. Hum Gene Ther. 2001;12:1917–1922. 11589833 10.1089/104303401753153965

[14] Mollenkopf HJ, Grode L, Mattow J, Stein M, Mann P, Knapp B, et al. Application of mycobacterial proteomics to vaccine design: improved protection by *Mycobacterium bovis* BCG prime-Rv3407 DNA boost vaccination against tuberculosis. Infect Immun. 2004;72:6471–6479. 15501778 10.1128/IAI.72.11.6471-6479.2004 PMC523041

[15] Saldaña A, Sousa OE. *Trypanosoma rangeli*: epimastigote immunogenicity and cross-reaction with *Trypanosoma cruzi* . J Parasitol. 1996;82:363–366. 8604121

[16] Stevens JR, Teixeira MM, Bingle LE, Gibson WC. The taxonomic position and evolutionary relationships of *Trypanosoma rangeli* . Int J Parasitol. 1999;29:749–757. 10404271 10.1016/s0020-7519(99)00016-8

[17] Grisard EC. Salivaria or Stercoraria? The *Trypanosoma rangeli* dilemma. Kinetoplastid Biol Dis. 2002;1:5 12234384 10.1186/1475-9292-1-5 PMC119326

[18] Guhl F, Vallejo GA. *Trypanosoma* (Herpetosoma) *rangeli* Tejera, 1920: an updated review. Mem Inst Oswaldo Cruz. 2003;98:435–442. 12937750 10.1590/s0074-02762003000400001

[19] Basso B, Cervetta L, Moretti E, Carlier Y, Truyens C. Acute *Trypanosoma cruzi* infection: IL-12, IL-18, TNF, sTNFR and NO in *T*. *rangeli*-vaccinated mice. Vaccine. 2004;22:1868–1872. 15121297 10.1016/j.vaccine.2003.11.013

[20] Basso B, Moretti E, Fretes R. Vaccination with epimastigotes of different strains of *Trypanosoma rangeli* protects mice against *Trypanosoma cruzi* infection. Mem Inst Oswaldo Cruz. 2008;103:370–374. 18660992 10.1590/s0074-02762008000400010

[21] Basso B, Castro I, Introini V, Gil P, Truyens C, Moretti E. Vaccination with *Trypanosoma rangeli* reduces the infectiousness of dogs experimentally infected with *Trypanosoma cruzi* . Vaccine. 2007;25:3855–3858. 17349724 10.1016/j.vaccine.2007.01.114 PMC7127752

[22] NOM-062-ZOO-1999. Especificaciones Técnicas para la Producción, Cuidado y Uso de los Animales del Laboratorio. 1999. Available from http://www.fmvz.unam.mx/fmvz/principal/archivos/062ZOO.PDF.

[23] NOM-033-ZOO-1995. Sacrificio Humanitario de los Animales Domésticos y Silvestre, 1995. Available from http://www.cuautitlan.unam.mx/descargas/cicuae/normas/Norma033.pdf.

[24] Camargo EP. Growth and Differentiation in *Trypanosoma cruzi*. I. Origin of Metacyclic Trypanosomes in Liquid Media. Rev Inst Med Trop Sao Paulo. 1964;6:93–100. 14177814

[25] Barbabosa-Pliego A, Díaz-Albiter HM, Ochoa-García L, Aparicio-Burgos E, López-Heydeck SM, Velásquez-Ordoñez V, et al. *Trypanosoma cruzi* circulating in the southern region of the State of Mexico (Zumpahuacan) are pathogenic: a dog model. Am J Tropl Med Hyg. 2009;81:390–395. 19706902 PMC2784919

[26] Murthy VK, Dibbern KM, Campbell DA. PCR amplification of mini-exon genes differentiates *Trypanosoma cruzi* from *Trypanosoma rangeli* . Mol Cell Probes. 1992;6:237–243. 1406732 10.1016/0890-8508(92)90022-p

[27] Untergasser A, Nijveen H, Rao X, Bisseling T, Geurts R, Leunissen JA. Primer3Plus, an enhanced web interface to Primer3. Nucleic Acids Res. 2007;35:71–74. 10.1093/nar/gkm306 PMC1933133 17485472

[28] Park HM. Univariate analysis and normality test using SAS, Stata, and SPSS The University Information Technology Services, Indiana University 2008;25.

[29] Stokes ME, Davis CS, Koch GG. Categorical data analysis using the SAS ® system. 2nd ed SAS Institute Inc., USA; 2000.

[30] Gupta S, Garg NJ. Prophylactic efficacy of TcVac2 against *Trypanosoma cruzi* in mice. PLoS Negl Trop Dis. 2010;4:e797 10.1371/journal.pntd.0000797 20706586 PMC2919396

[31] Gupta S, Wan X, Zago MP, Sellers VC, Silva TS, Assiah D, et al. Antigenicity and diagnostic potential of vaccine candidates in human Chagas disease. PLoS Negl Trop Dis. 2013;7:e2018 10.1371/journal.pntd.0002018 23350012 PMC3547861

[32] Gupta S, Garg NJ. TcVac3 induced control of *Trypanosoma cruzi* infection and chronic myocarditis in mice. PLOS ONE. 2013;8:e59434 10.1371/journal.pone.0059434 23555672 PMC3608676

[33] Basso B, Moretti ER, Vottero-Cima E. Immune response and *Trypanosoma cruzi* infection in *Trypanosoma rangeli*-immunized mice. Am J Trop Med Hyg. 1991;44:413–419. 1828328 10.4269/ajtmh.1991.44.413

[34] Cervetta L, Moretti E, Basso B. Experimental Chagas’ disease: the protection induced by immunization with *Trypanosoma rangeli* is associated with down-regulation of IL-6, TNF-alpha and IL-10 synthesis. Acta Parasitol. 2002;47:73–78.

[35] Rodríguez-Morales O, Carrillo-Sánchez SC, García-Mendoza H, Aranda-Fraustro A, Ballinas-Verdugo MA, Alejandre-Aguilar R, et al. Effect of the plasmid-DNA vaccination on macroscopic and microscopic damage caused by the experimental chronic *Trypanosoma cruzi* infection in the canine model. BioMed Res Int. 2013;8:65–70. 10.1155/2013/826570 PMC3791577 24163822

[36] Bergeron LM, McCandless EE, Dunham S, Dunkle B, Zhu Y, Shelly J, et al. Comparative functional characterization of canine IgG subclasses. Vet Immunol Immunopathol. 2014;157:31–41. 10.1016/j.vetimm.2013.10.018 24268690

[37] Guedes PM, Veloso VM, Gollob KJ, Afonso LC, Caldas IS, Vianna P, et al. IgG isotype profile is correlated with cardiomegaly in Beagle dogs infected with distinct *Trypanosoma cruzi* strains. Vet Immunol Immunopathol. 2008;124:163–168. 10.1016/j.vetimm.2008.03.003 18439688

[38] Hamilton PB, Lewis MD, Cruickshank C, Gaunt MW, Yeo M, Llewellyn MS, et al. Identification and lineage genotyping of South American trypanosomes using fluorescent fragment length barcoding. Infect Genet Evol. 2011;11:44–51. 10.1016/j.meegid.2010.10.012 21029792

[39] Slauson DO, Cooper BJ. Mechanisms of disease: a textbook of comparative general pathology: 3rd ed Mosby Inc., St. Luis; 2002.

